# Preventing adolescent synaptic pruning in mouse prelimbic cortex via local knockdown of α4βδ GABA_A_ receptors increases anxiety response in adulthood

**DOI:** 10.1038/s41598-021-99965-8

**Published:** 2021-10-26

**Authors:** Matthew R. Evrard, Michael Li, Hui Shen, Sheryl S. Smith

**Affiliations:** 1 Department of Physiology and Pharmacology, SUNY Downstate Medical Center, 450 Clarkson Ave., Brooklyn, NY 11203 USA; 2 Graduate Program in Neural and Behavioral Science, SUNY Downstate Medical Center, 450 Clarkson Ave., Brooklyn, NY 11203 USA; 3 College of Arts and Sciences, Hunter College, New York, NY 10065 USA; 4 School of Biomedical Engineering, Tianjin Medical University, No. 22 Qixiangtai Road, Heping District, Tianjin, 300070 China

**Keywords:** Emotion, Ion channels in the nervous system, Synaptic plasticity

## Abstract

Anxiety is increasingly reported, especially in adolescent females. The etiology is largely unknown, which limits effective treatment. Layer 5 prelimbic cortex (L5PL) increases anxiety responses but undergoes adolescent synaptic pruning, raising the question of the impact of pruning on anxiety. Here we show that preventing L5PL pruning increases anxiety in response to an aversive event in adolescent and adult female mice. Spine density of Golgi-stained neurons decreased ~ 63% from puberty (~ PND35, vaginal opening) to post-puberty (PND56, P < 0.0001). Expression of α4βδ GABA_A_ receptors (GABARs) transiently increased tenfold in L5PL at puberty (P < 0.00001), but decreased post-pubertally. Both global and local knockdown of these receptors during puberty prevented pruning, increasing spine density post-pubertally (P < 0.0001), an effect reversed by blocking NMDA receptors (NMDARs). Pubertal expression of the NMDAR-dependent spine protein kalirin7 decreased (50%, P < 0.0001), an effect prevented by α4 knock-out, suggesting that α4βδ-induced reductions in kalirin7 underlie pruning. Increased spine density due to local α4 knockdown at puberty decreased open arm time on the elevated plus maze post-pubertally (62%, P < 0.0001) in response to an aversive stimulus, suggesting that increases in L5PL synapses increase anxiety responses. These findings suggest that prelimbic synaptic pruning is necessary to limit anxiety in adulthood and may suggest novel therapies.

## Introduction

More people suffer from anxiety than any other mental disorder^1^. While anxiety is a normal physiological state necessary to avoid danger, chronic anxiety results in maladaptive and excessive avoidance, which can interfere with cognitive functioning and quality of life^1^. Since their initial classification as mental disorders in the 1980s, anxiety disorders have out-paced all others^1,2^. The most recent estimates suggest that, in less than a decade, anxiety has increased by 40% in adults^3^ and by as much as 75% in adolescents^4^. Anxiety disorders are most likely to develop in adolescence^1,5^, but left unrecognized, are the root of adult anxiety^6^. This is especially relevant now as many reports indicate a substantial increase due to the Covid-19 pandemic^7^. Anxiety is especially pronounced in response to stressful, aversive stimuli^8,9^. However, this disorder's etiology is unknown at the circuit level, and approximately half of the affected individuals do not receive effective treatment^10^. Thus, gaining insight into the CNS substrates that are vulnerable during adolescence would lead to more effective therapies to reduce anxiety across the lifespan.

The brain circuits involved in anxiety expression include the medial prefrontal cortex (mPFC), amygdala, and ventral hippocampus^11^. Sub-regions of the mPFC include the prelimbic (PL), which has excitatory projections to the amygdala and is associated with negative emotions such as fear and anxiety^12^ which are triggered by an aversive stimulus^13,14^. Lesions and pharmacological inactivation of the PL reduce anxiety in rodents^15^, while activation of this region increases anxiety^16^. In contrast, the adjacent infralimbic mPFC (IL) has inhibitory control over the amygdala^17^ and is associated with fear extinction^12^ and decreased expression of anxiety^18^.

Activity of the output layer 5 (L5) neurons in the PL can generate anxiety behavior which is dependent upon a critical density of the dendritic spines that receive long-range and short-range excitatory synaptic inputs^19^. At puberty, dendritic spines and grey matter in the human and non-human mPFC decrease by half (“synaptic pruning”)^20,21^, which, in the human, is paralleled by EEG changes linked to pubertal maturation^22^. The PL, specifically, has been shown to undergo extensive synaptic pruning^23^ in adolescence, while the adjacent IL does not^23^. However, the role of adolescent PL pruning in regulating anxiety behavior is not yet known, nor are the consequences of reduced pruning in adolescence, which would produce increased excitatory input to this area.

This study addressed this issue by assessing dendritic spine density in L5 PL after the onset of puberty (~ PND 35, assessed by vaginal opening) compared with post-puberty (PND 56). We examined these changes in female mice because anxiety is most likely to afflict females^2^. To manipulate pruning in this area, we first examined a potential mechanism, an atypical GABA_A_ receptor (GABAR), α4βδ^24^, which expresses on dendritic spines at puberty as well as along the dendritic shaft and on the soma in some CNS regions to inhibit synaptic input. In contrast to typical GABARs, which express post-synaptically to GABAergic interneurons, α4βδ GABARs express away from GABAergic synapse, have a high sensitivity to ambient GABA, which is maintained by GABA transporters^25^, and display little desensitization^26^.

Inhibition impairs the activation of NMDA receptors^24^, which are necessary for spine maintenance^27^ via regulation of kalirin-7 (Kal-7), a spine protein that stabilizes the actin cytoskeleton^28^. Thus, increases in inhibition generated by increased α4βδ GABARs in L5 PL at puberty is a potential mechanism for synapse pruning of this region as shown for other CNS areas^29^.

For the present study, we examined the role of α4βδ receptors in mediating pubertal synaptic pruning using both pharmacological and genetic tools. Selective deletion of these receptors in PL at puberty using viral delivery of Cre recombinase to a mouse with loxP (locus of X-over P1) sites flanking the α4 gene allowed us to determine if a high spine density in the PL, in the absence of pruning, increases anxiety-like behavior. We used the shock-paired elevated plus maze (EPM) to assess avoidance, which has been validated as a measure of anxiety level in humans^30^ and to more closely compare with clinical studies showing PL activation triggered by an aversive event^13,14^ Additional experiments investigated the role of α4βδ impairment of NMDAR activation and subsequent reduction in Kal-7 levels at puberty on synaptic pruning. Understanding the role of α4βδ GABARs in mPFC pruning during adolescence is highly relevant for understanding mechanisms that underlie mental disorders such as anxiety and depression, where abnormal expression of α4 and/or δ has been reported^31,32^.

## Results

### Pyramidal cells in L5 PL of the female mouse undergo synaptic pruning

**Figure 1:**
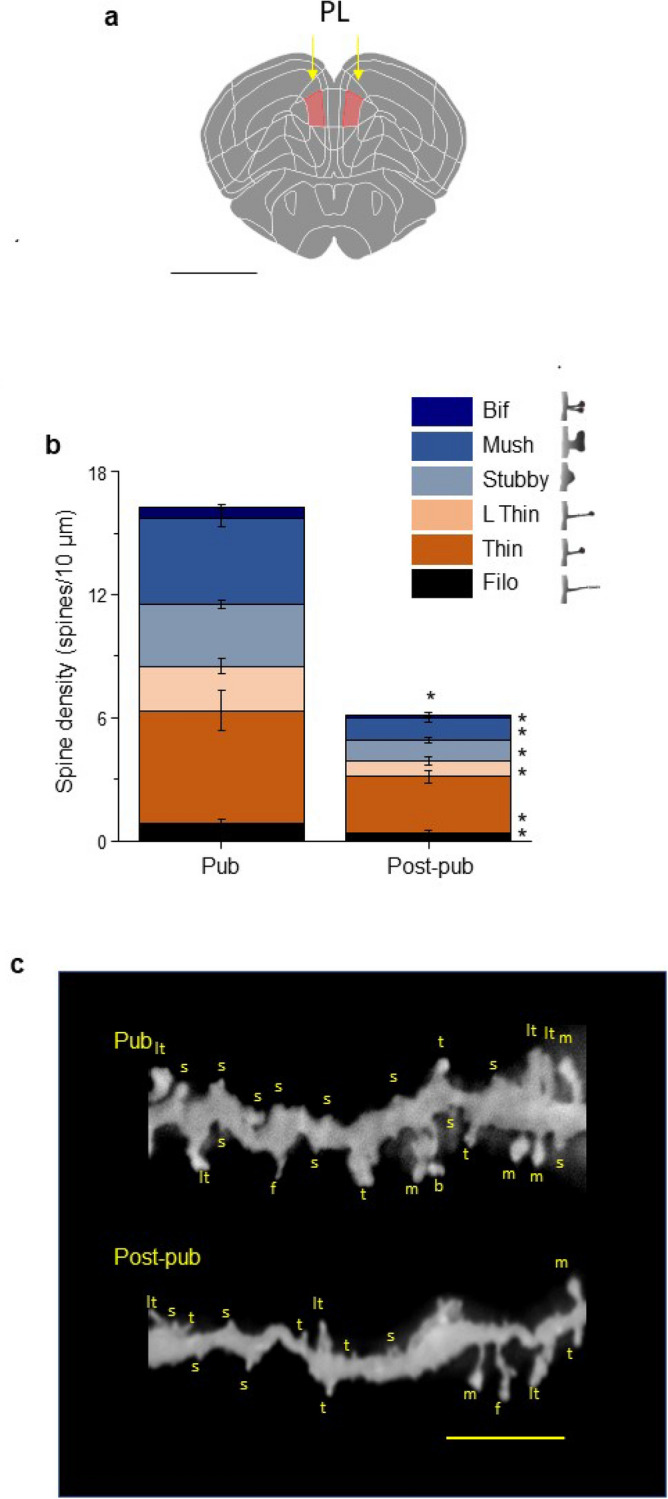
Spine density in layer 5 prelimbic cortex (L5 PL) of the female mouse decreases by half during adolescence: assessment of spine-types. (**a**) Schematic diagram of prelimbic cortex (PL) localization in mouse brain (pink shading, arrows). Coronal section, 2.09 mm anterior to Bregma. Scale, 1 mm. (**b**) Averaged data, spine density (#spines/10 μm) of layer 5 (L5) PL for pubertal (Pub, ~ PND 35, assessed by vaginal opening) and post-pubertal (Post-pub, PND 56) female mice. Total spines, t(20) = 6.43, *P < 0.0001; bifurcated, t(20) = 3.71, *P = 0.0014; mushroom, t(20) = 6.2, *P < 0.0001; stubby, t(20) = 6.18, *P < 0.0001; long thin, t(20) = 3.46, *P = 0.0025; thin, t(20) = 2.79, *P = 0.0114; filopodia, t(20) = 2.24, *P = 0.037. (**c**) Representative images of basal dendrites from Golgi-stained neurons, from Pub and Post-pub female mice. Spine-types: *f* filopodia, *lt* long thin, *t* thin, *s* stubby, *m* mushroom. Scale, 5 μm. n = 43–44 neurons, 11 mice/group.

Spine density of the basilar dendrites of L5 PL pyramidal cells (see Fig. 1a for localization) was determined by microscopic examination of z-stacks of Golgi-stained neurons. Averaged values of spine density across the dendrite decreased by ~ 63% from puberty onset (~ PND 35) to PND 56 (Fig. 1b,c, 16.39 ± 1.55 spines/10 μm, pub vs. 6.10 ± 0.58 spines/10 μm, post-pub, P < 0.0001). The greatest decline (71%, P < 0.0001) was for the spines considered stable in terms of their long-lasting presence (mushroom, stubby and bifurcated). Of the commonly noted spine-types, the mushroom spines exhibited the greatest decline (74%, P < 0.0001); the stubby spines also decreased significantly (66%, P < 0.0001), as did the relatively rare bifurcated spines (84%, P = 0.0014). The density of the less stable (motile) spines decreased by 53%, which included significant decreases in long, thin (64%, P = 0.0025), and thin (49%, P = 0.0114) spines.

### α4βδ GABAR expression increases transiently at the onset of puberty in L5 PL

**Figure 2:**
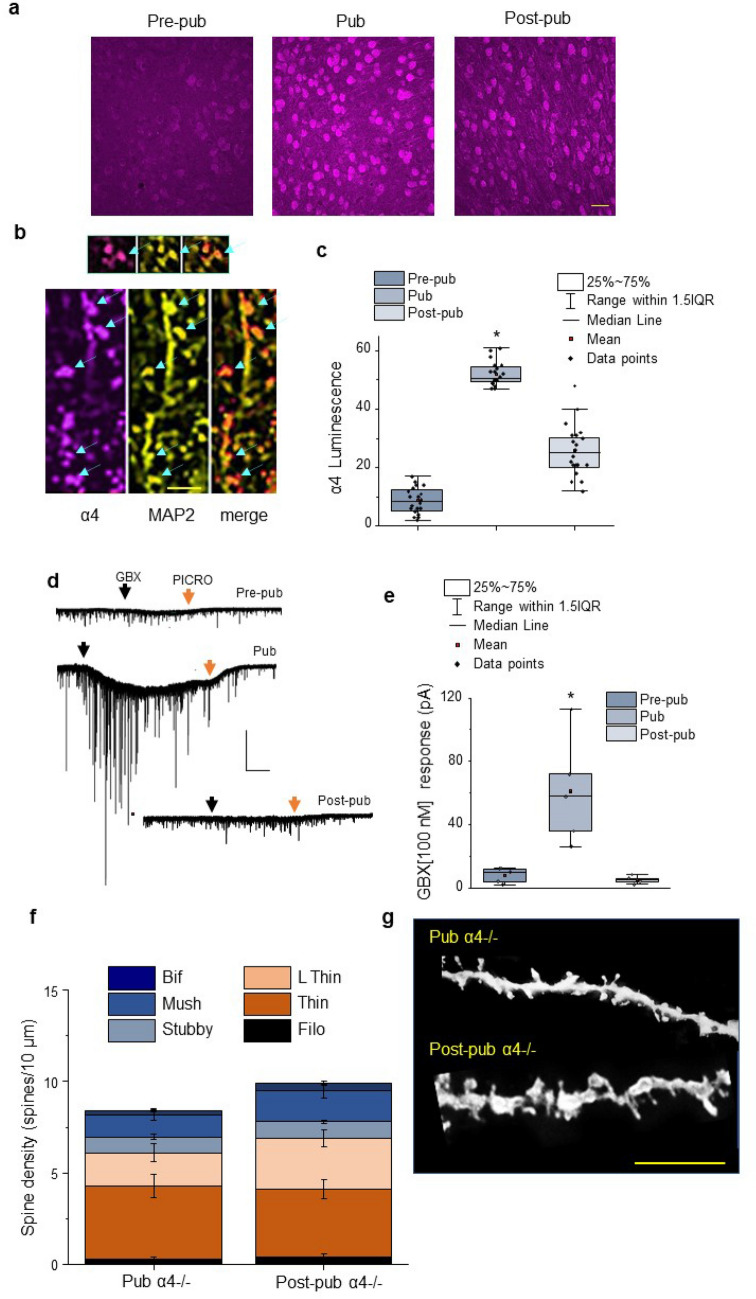
Increases in α4βδ GABAR expression in L5 PL at puberty are necessary for synaptic pruning in female mice. (**a**) Representative images, α4 immunostaining (magenta), L5 PL pyramidal cells from pre-pubertal (pre-pub, left), pubertal (pub, center), and post-pubertal (post-pub, right) female mice (α4, magenta). Scale, 100 μm. Z-stack sequences used for merged images are presented in Supp. Figure 1. (**b**) Representative images, α4 (magenta, left), MAP2 (yellow, middle), and merged (orange, right) on the dendrites and spines (arrows) of pyramidal cell in L5 PL. from a pubertal mouse. Inset, Co-localization of α4 and MAP2 on a spine (arrow). Scale, 2 μm. (**c**) Averaged data, mean, median, and interquartile range (IQR). F(2,57) = 248.9, P < 0.00001, *P < 0.05 vs. other groups. n = 15 neurons, 10 mice/group. (**d**) Representative whole cell voltage-clamp recordings of L5 PL pyramidal cell response to 100 nM gaboxadol (GBX, black arrow) from Pre-pub, Pub, and Post-pub female mice. PICRO (picrotoxin, 100 μM, red arrow). Scale, 50 s, 200 pA. (**e**) Averaged data, mean, median, interquartile range, and individual data points for GBX responses. F(2,12) = 12.28, *P = 0.00125. *P < 0.05 vs. other groups. n = 5 mice/group. (**f**) Averaged data, spine density (#spines/10 μm) of L5 PL from a Pub and Post-pub female α4^−/−^ mouse. (**g**) Representative images, Golgi-stained dendrites from pub and post-pub female α4^−/−^ mice. Scale, 5 μm. n = 13–31 neurons, 8 mice/group.

Extrasynaptic α4βδ GABARs increase at puberty in some brain areas when they express on soma, along the dendritic shaft and spine^24^. Therefore, we initially determined whether these receptors increase at puberty in L5 PL. α4 expression was assessed using immunohistochemical techniques before puberty (~ PND 28–32), just after puberty onset (~ PND 35), and post-pubertally (PND 56). Immunostaining for α4 increased almost tenfold at the onset of puberty compared to pre-puberty (Fig. 2a,c, Supp. Figure 1, P < 0.00001) and then declined ~ 75% post-pubertally. Additional studies co-localizing α4 immunostaining with microtubule-associate protein-2 (MAP2), a protein that expresses in mushroom spines^33^, reveal that α4 immunostaining is localized to both the dendrite and the dendritic spine (Fig. 2b) in addition to the cell body.

In order to confirm increased expression of functional α4βδ GABARs at puberty, we assessed the response of L5 PL neurons to gaboxadol (GBX), a GABA agonist which is selective for α4βδ GABARs at a concentration of 100 nM^26,34^. When applied in vitro, the relative neuronal responses can be used as a functional index of α4βδ GABAR expression^24^. To this end, L5 PL pyramidal cells were recorded in the slice preparation from pre-pubertal, pubertal, and post-pubertal female mice using whole-cell voltage clamp techniques. Application of 100 nM GBX elicited a tenfold greater response at puberty than pre-puberty and post-puberty (Fig. 2d,e,  P = 0.00125), suggesting that functional α4βδ GABARs increase transiently at puberty in L5 PL.

### Synaptic pruning of L5 PL pyramidal cells is prevented by knock-out of the GABAR α4 subunit

The increase in α4βδ GABARs on L5 PL pyramidal cells at puberty raised the possibility that these receptors may play a role in L5 PL synaptic pruning. Thus, we assessed spine density in pubertal and post-pubertal α4^−/−^ female mice. Unlike the wild-type mouse, total spine density did not significantly change from puberty to post-puberty in L5 PL pyramidal cells from the global α4 knock-out mouse (Fig. 2f,g), nor did spine density for any of the different spine types assessed.

### Adolescent synaptic pruning of male L5 PL is a result of increased α4βδ GABAR expression

We also investigated synaptic pruning in the male L5 PL. Spine density in the male L5 PL decreased by ~ 53% in adolescence (P < 0.0001) with significant decreases in stubby, thin and long, thin spines (Supp. Figure 2, P < 0.03) while expression of the GABAR α4 subunit in L5 PL pyramidal cells increased sixfold (P < 0.00001) at puberty (Supp. Figure 2) compared to pre-puberty, before declining 75% by PND 56 (P < 0.00001). Total spine density did not change significantly during adolescence in the male α4^−/−^ mouse (Supp. Figure 2), although there was a significant (P = 0.0076) 59% decrease in the long, thin spines.

### Effects of pharmacological manipulation of GABARs on synaptic pruning

**Figure 3:**
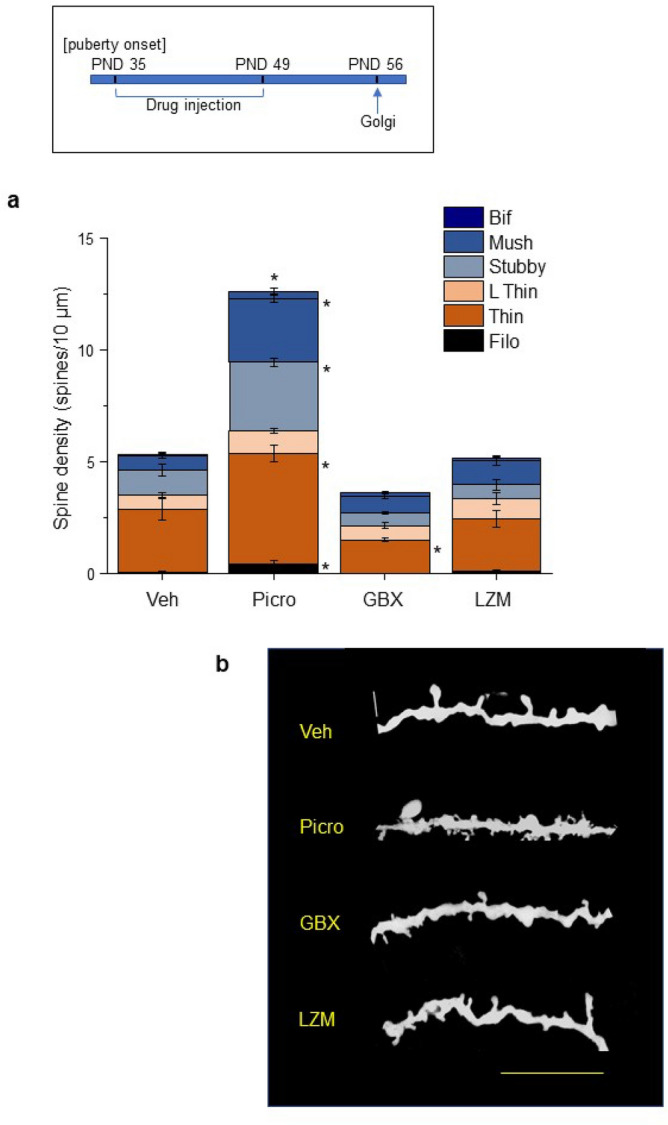
Pharmacological manipulation of GABAR-gated current during puberty alters spine density post-pubertally. Inset, timeline of drug administration during the pubertal period of high α4 expression (PND 35–49) using picrotoxin (PICRO, 3 mg/kg, i.p.), gaboxadol (GBX, 0.1 mg/kg,i.p.), lorazepam (LZM, 0.25 mg/kg, i.p.) or vehicle (VEH). Mice were euthanized for Golgi procedures on PND 56. (**a**) Averaged data, Total spines, F(3,22) = 119.5, P < 0.0001 [Veh vs. Picro, *P < 0.0001]; mushroom, F(3,22) = 25.53, P < 0.0001 [Veh vs. Picro, *P < 0.0001]; stubby, F(3,22) = 43.76, P < 0.0001 [Veh vs. Picro, *P < 0.0001]; thin, F(3,22) = 15.45, P < 0.0001 [Veh vs. Picro, *P = 0.0019; Veh vs. GBX, *P = 0.0445]; filopodia, F(3,22) = 4.71, P = 0.011 [Veh vs. Picro, *P < 0.02]. (**b**) Representative images of basal dendrites from Golgi-stained neurons from Post-pub female WT mice treated with the indicated drugs during puberty. Scale, 10 μm. n = 14–23 neurons, 7 mice/group.

We tested whether systemic pharmacological manipulation of GABARs at the circuit level during the pubertal period (PND 35-49) alters PL spine density at PND 56. Systemic administration of the non-selective GABAR antagonist picrotoxin, at a dose sub-threshold for seizure induction, for 2 weeks beginning at the onset of puberty (~ PND 35, 3 mg/kg, i.p., daily, Fig. 3a inset) increased spine density by ~ 3.5-fold compared to vehicle (P < 0.0001, Fig. 3a,b) on PND 56, with greatest increases (almost threefold, P < 0.0001) in the mushroom spines. Significant increases in the filopodia (P = 0.011), thin spines (P = 0.0019), and stubby spines (P < 0.0001) were also observed, suggesting a role of GABARs in PL pruning.

In order to potentiate current gated by α4βδ GABARs during puberty, GBX was injected across the same pubertal period at a dose (0.1 mg/kg, i.p.) selective for α4βδ GABARs^35^. Augmenting α4βδ inhibition in this way produced a 47% decrease in density of the thin spines on PND 56 (P = 0.045, Fig. 3a,b), confirming that α4βδ GABARs, at the network level, play a role in adolescent pruning of L5 PL.

In the cortex, GABAergic interneurons have synaptic contacts on dendritic spines^36^, which activate GABARs containing γ2 rather than δ, raising the possibility that pruning is altered by these receptors. Thus, we injected lorazepam (LZM, 0.25 mg/kg, i.p.), a benzodiazepine positive allosteric modulator of the GABAR subtypes found at synaptic sites containing α(1-3,5)βxγ2^37^. LZM administration across the pubertal period did not alter spine density (Fig. 3a,b). Taken together, these data suggest that α4βδ GABARs trigger synaptic pruning in L5 PL, and that synaptic α1βγ2 GABARs do not contribute to this process.

### Role of NMDARs in synaptic pruning of L5 PL

**Figure 4:**
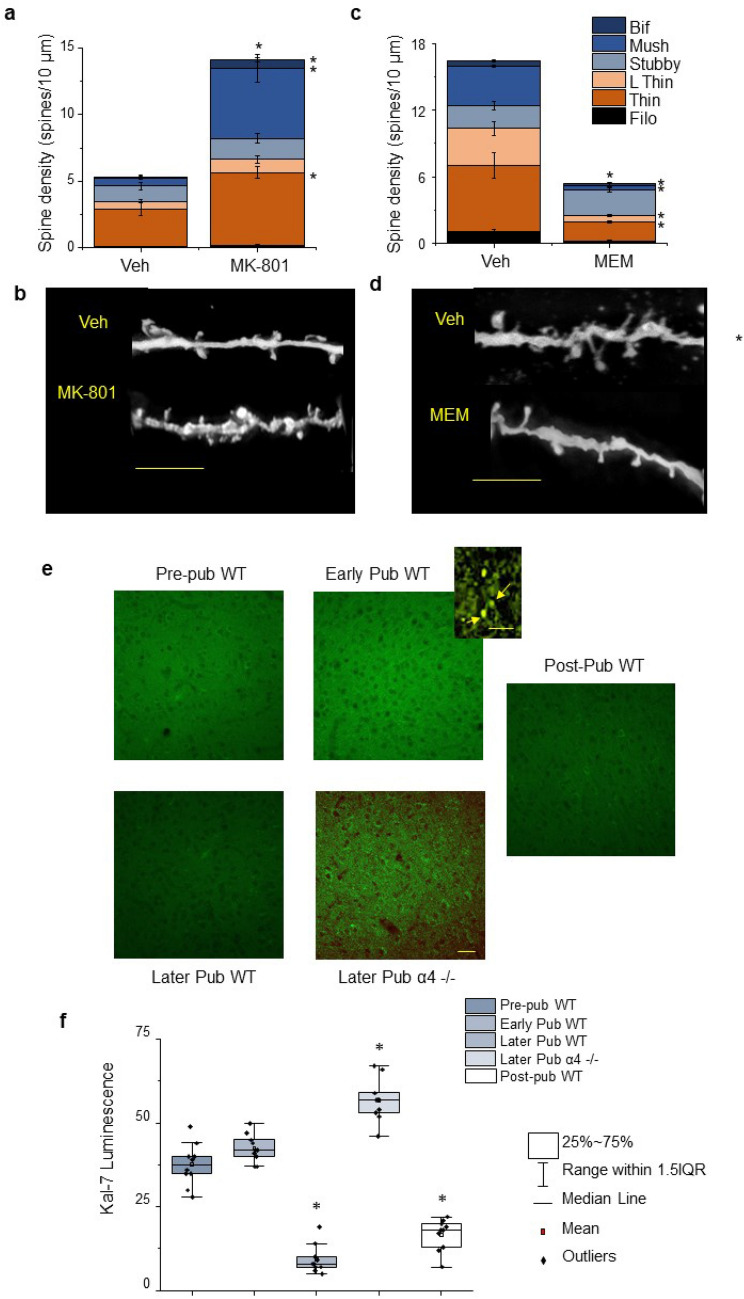
Pubertal state and α4 knock-out: Effects on NMDA receptor-regulated pruning and expression of the spine protein kalirin-7. (**a**) Averaged data, spine density (#spines/10 μm) of post-pubertal (PND 56) female WT mice treated during the pubertal period (PND 35–49, inset) with MK-801 (0.1 mg/kg, i.p.) which paradoxically increases NMDA receptor expression in mPFC^34^ (Supp. Figure 3). Total spines, t(12) = 5.32, *P = 0.0002; bifurcated, t(12) = 4.16, *P = 0.0002; mushroom, t(12) = 4.53, *P = 0.0007; stubby, t(12) = 1.1, P = 0.206; thin, t(12) = 3.76, *P = 0.0027; long thin, t(12) = 1.25, *P = 0.0027. (**b**) Representative images of basal dendrites from Golgi-stained neurons, from the indicated groups. Scale, 5 μm. n = 18–21 neurons, 7 mice/group. (**c**) Averaged data, spine density of post-pubertal female α4^−/−^ mice treated during the pubertal period (PND 35–49) with memantine (0.1 mg/kg, i.p.) an NMDA receptor antagonist. Total spines, t(18) = 4.54, *P = 0.0003; bifurcated, t(18) = 2.6, *P = 0.018; mushroom, t(18) = 3.87, *P = 0.0011; long thin, t(18) = 4.53, *P = 0.0003; thin, t(18) = 3.69, *P = 0.0017; filopodia, t(18) = 5.46, *P < 0.0001. (**d**) Representative images of basal dendrites from Golgi-stained neurons, from the indicated groups. Scale, 5 μm. n = 29–41 neurons, 10 mice/group. (**e**) Representative images, kalirin-7 (Kal-7, green) immunostaining of L5 PL from pre-pubertal (pre-pub), early pubertal (early pub, day of vaginal opening, later pubertal (later pub) and post-pubertal (post-pub) WT female mice as well as from a pubertal α4^−/−^ mouse (Later Pub α4^−/−^). Scale, 100 μm. Inset, spine localization of Kal-7. Scale, 4 μm. (Z-stack sequences used for merged images are presented in Supp. Figure 4). (**f**) Averaged data, mean, median and interquartile range (IQR) from the indicated groups. F(4,45) = 139, P < 0.0001; *P < 0.05 vs. other unstarred groups. n = 20 neurons, 9 mice/group.

α4βδ GABARs impair the activation of NMDARs^24^, which maintain dendritic spine stability^27^. Thus, we tested whether over-expressing NMDARs during the pubertal increase in α4βδ expression (~ PND 35–49) would increase spine density on PND 56. Administration of a low dose of MK-801 (0.1 mg/kg, i.p.), paradoxically increases NMDAR expression by more than twofold in L5PL after 5 days (P < 0.0001, Supp. Figure 3) as a compensatory response^38^. MK-801 treatment during the pubertal period prevented adolescent pruning in wild-type mice (Fig. 4a,b), similar to α4 knock-out, resulting in spine densities which were almost threefold greater than the PND 56 vehicle control (P = 0.0002). The greatest increase in commonly observed spine types was for the mushroom spines, which increased sevenfold above control levels (P = 0.0007), while the thin spines increased by twofold (P = 0.0027). The bifurcated spines were also significantly increased (P = 0.0002).

We then used the NMDAR blocker memantine, which does not increase NMDAR expression^39^ due to its high binding affinity^40^. Blockade of NMDARs during puberty restored pruning in α4^−/−^ mice which previously exhibited impaired pruning (Fig. 4c,d). This treatment resulted in spine densities of approximately one-third those of the α4^−/−^ control (P = 0.0003) at PND 56. All spine-type densities were significantly lower after memantine treatment except for the stubby spines, with the greatest effects on the mushroom spines (P = 0.001), long thin spines (P = 0.0003), and filopodia (P < 0.0001). These data suggest that NMDAR activity plays a role in synaptic pruning of L5 PL.

### Expression of the spine protein Kal-7 decreases at puberty in wild-type but not α4^−/−^ L5 PL

The spine protein Kal-7 is necessary for spine maintenance^28^. Therefore, we assessed expression levels of this protein in L5 PL of wild-type and α4^−/−^ mice before puberty, during puberty, and post-pubertally with immunohistochemical techniques. Kal-7 expression in L5 PL was unchanged at the onset of puberty (vaginal opening, PND 35) but decreased 75% by PND 40 (P < 0.0001) compared to pre-pubertal values (Fig. 4e,f, Supp. Figure 4), assessed in wild-type mice. However, Kal-7 expression levels partially recovered on PND 56, increasing by almost twofold (P < 0.0001), suggesting an inverse correlation with α4 expression. Kal-7 expression in pubertal (PND 36–40) α4^−/−^ mice was fivefold greater than comparable expression in pubertal wild-type mice of the same age (P < 0.0001), implicating α4βδ receptors as the cause for the decline in Kal-7 expression at puberty.

### Local pubertal α4 knockdown increases PL spine density at PND 56

**Figure 5:**
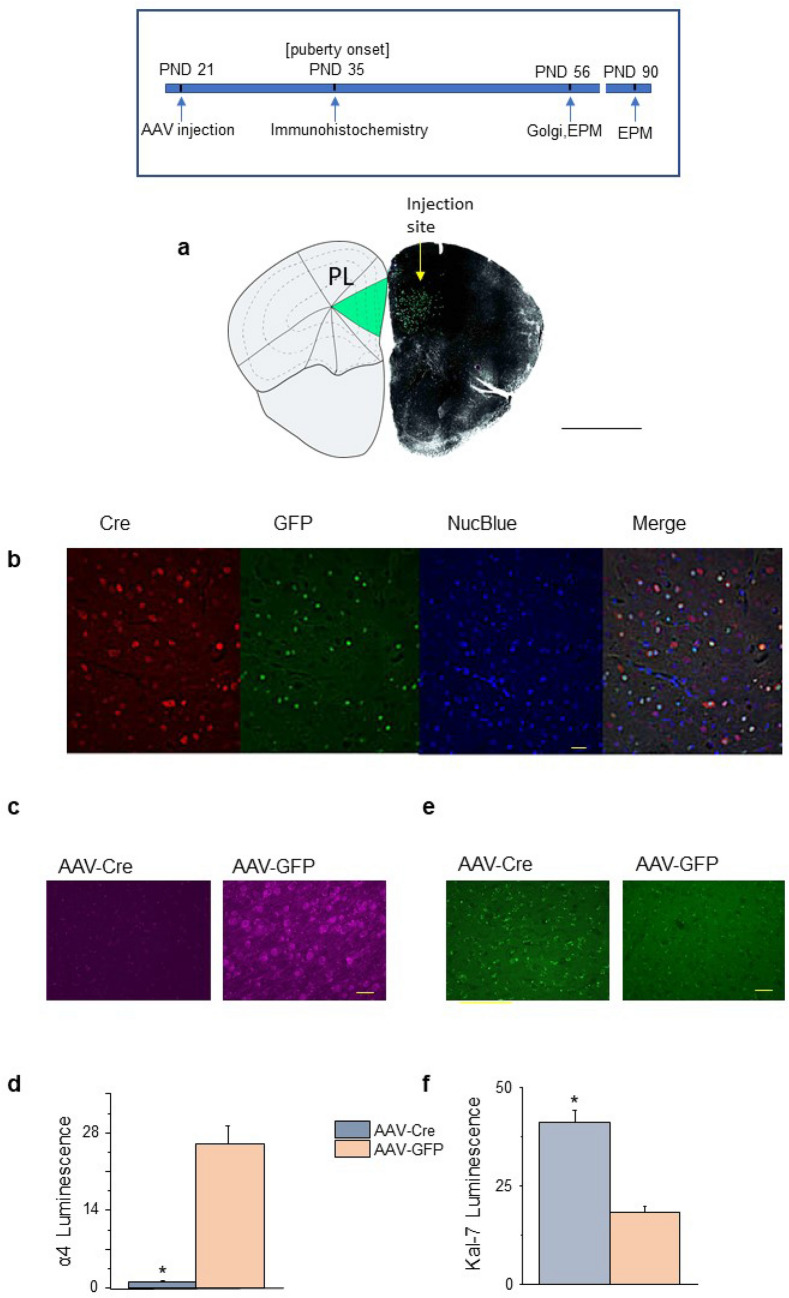
Local infusion of AAV-Cre into PL on PND 21 results in Cre expression and α4 knockdown in PND 35 female mice. Inset, Timeline showing the day of AAV injection (PND 21) when transgenic female mice expressing LoxP sites flanking the GABRA4 gene were injected bilaterally into the PL with adeno-associated virus (AAV)-Cre recombinase or AAV-green fluorescent protein (GFP) and testing (PND 35, α4 immunohistochemistry; PND 56, Golgi spine protocol; PND 56 and 90, anxiety tested using the EPM). (**a**) Left, schematic showing PL (green). Right, Representative image at PND 35 of GFP staining restricted to the PL (green) boundaries following injection of AAV-Cre/GFP at PND 21. Scale, 2067 μm. This coronal slice was taken 1.767 μm anterior to Bregma. (**b**) Representative images (×40) at PND 35 following injection of AAV-Cre/GFP at PND 21 of Cre recombinase (Cre) immunostaining (left to right, Cre, GFP, nuclear blue (Nuc blue), merged); Scale, 50 μm. (**c**) Representative images of α4 immunostaining at PND 35 after infusion of AAV-Cre or AAV-GFP on PND 21 . Scale, 100 μm. (Z-stack sequence, Supp. Figure 5). (**d**) Averaged data, t(18) = 7.27, *P < 0.00001. n = 10 neurons, 5 mice/group. (**e**) Representative images of Kal-7 immunostaining after infusion of AAV-Cre or AAV-GFP on PND 21. Scale, 100 μm. (Z-stack sequence, Supp. Figure 6). (**f**) Averaged data, t(18) = 6.36, *P < 0.00001. n = 10 neurons, 4 mice/group.

Because the α4 global knock-out is not selective for the PL, stereotaxic virus injections were used to selectively knockdown α4 in the PL at puberty to confirm its role in pruning: AAV-Cre or AAV-GFP (control) was injected into the PL of PND 21 transgenic mice with loxP sites flanking the α4 gene. Immunohistochemical analysis verified that the infusions targeted the PL (Fig. 5a) and induced Cre recombinase (Fig. 5b) by PND 35. α4 staining in L5 PL was almost undetectable in the AAV-Cre group on PND 35 compared to the robust staining of the AAV-GFP group, which was 26-fold greater than for the AAV-Cre group (P < 0.00001, Fig. 5c,d, Supp. Figure 5a), assessed after puberty onset (PND 35–38) demonstrating successful α4 knockdown. In contrast, α4 staining was not altered in the IL (Supp. Figure 5b). Local knock-down of α4 using local AAV-Cre infusion was also associated with a ~ two-fold increase in expression of Kal-7 (Fig. 5e,f, Supp. Figure 6, P < 0.00001) compared to the GFP control, suggesting that α4βδ GABARs regulate Kal-7 expression.

**Figure 6:**
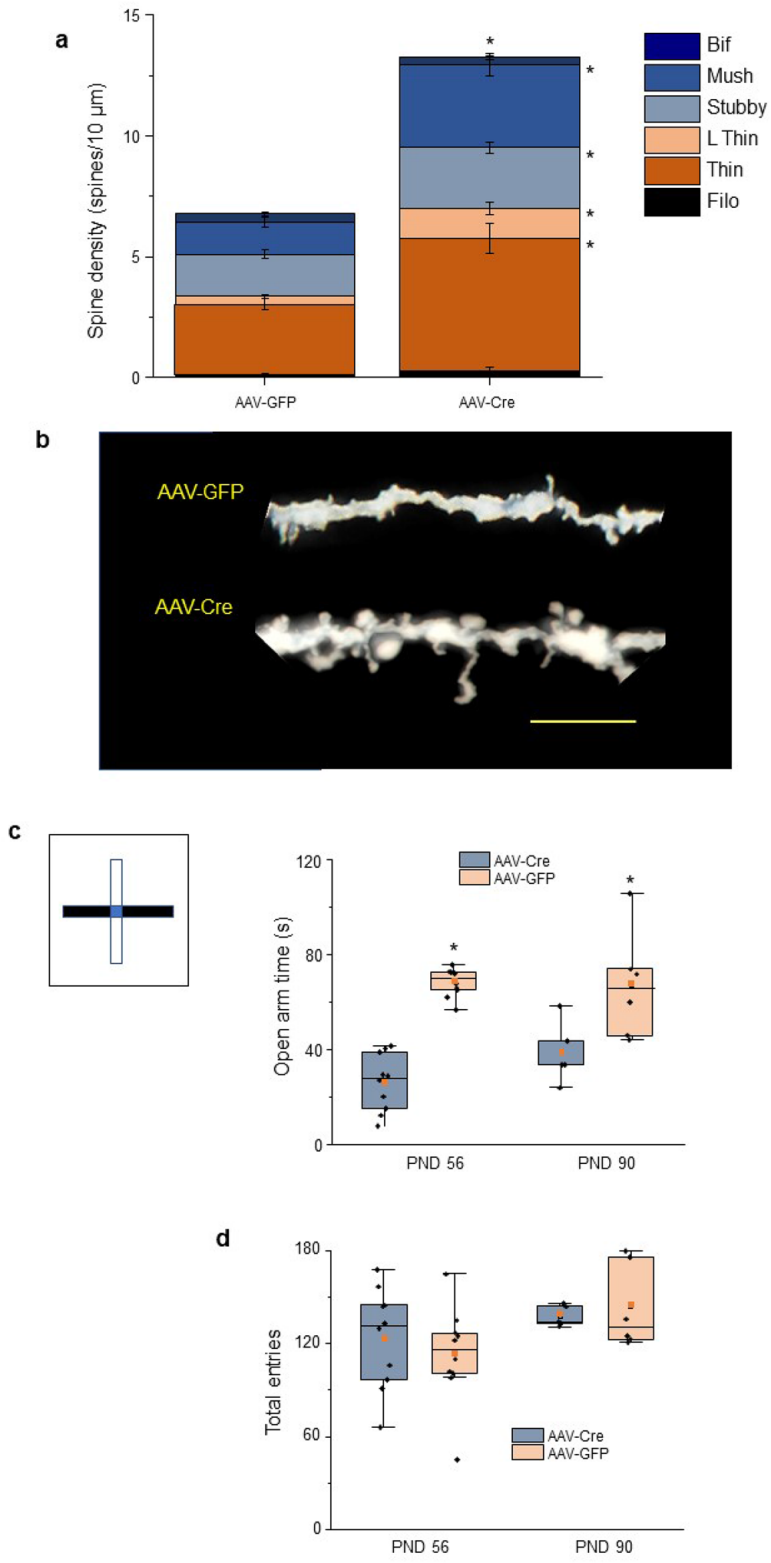
Local α4 knockdown at puberty increases spine density of L5 PL pyramidal cells at PND 56 and increases the anxiety response to an aversive stimulus. (**a**) Averaged data, spine density (#spines/10 μm) in post-pubertal (PND 56) female L5 PL following local α4 knockdown with AAV-Cre (right) compared to the AAV-GFP control (left). Total spines, t(16) = 4.94, *P = 0.0001; mushroom, t(16) = 3.89, *P = 0.0013; stubby, t(16) = 2.38, *P = 0.03; thin, t(16) = 3.63, *P = 0.0023; long thin, t(16) = 3.13, *P = 0.0065. (**b**) Representative images of basal dendrites from Golgi-stained neurons from the indicated groups. Scale, 5 μm. n = 36 neurons, 9 mice/group. (**c**) Inset, schematic drawing of elevated plus maze (EPM; arms, 30 cm long, 5 cm wide, elevated 40 cm): white, open arms, half enclosed by walls 7 cm high, extending 15 cm from the center; black, closed arms enclosed by walls 15.25 cm high for the entire length of the arm. (**c**) Mean (red square), median line, 25–75% interquartile range (IQR), SEM, and individual data points for open arm time in the EPM for PND 56 or PND 90 female mice injected with AAV-GFP or AAV-Cre in the PL on PND 21. PND 56, t(18) = 9.97, *P < 0.00001; PND 90, t(9) = 2.45, *P = 0.0366. A decrease in open arm time is an increase in avoidance behavior which reflects anxiety. (**d**) Mean (red square), median line, 25–75% interquartile range (IQR), SEM, and individual data points for total crossings in the EPM, measuring locomotor activity. n = 10 mice/group (PND 56); n = 5–6 mice/group (PND 90).

Local pubertal α4 knockdown increased spine density of L5 PL ~ twofold at PND 56 compared to the GFP control (P < 0.0001, Fig. 6a,b). Increases of similar magnitude were seen for the stable and motile spine types in L5 PL after local α4 knockdown compared to the GFP control. Mushroom spines exhibited the greatest increase, more than 2.5-fold (P = 0.0013), after local α4 knockdown, while all other spine types, except for bifurcated and filopodia, also increased. These findings suggest that the high expression of extrasynaptic α4βδ GABARs at puberty in PL triggers synapse loss during adolescence.

### Local knockdown of α4βδ GABARs in PL during puberty increases anxiety responses to an aversive stimulus post-pubertally

In order to determine the behavioral consequence of increased spine density in L5 PL as a result of reduced pubertal pruning in the absence of α4 expression, we assessed avoidance behavior post-pubertally at PND 56 as well as in adulthood (PND 90) following local α4 knockdown in the PL during puberty (Fig. 5a inset). The PL has been shown to increase anxiety responses^12^, which can be assessed by measuring avoidance behavior on the elevated plus-maze (EPM, Fig. 6c inset). Decreased time spent on the open arm reflects an increase in anxiety-like behavior. Shock-pairing was used to increase the paradigm's aversive quality, as we have demonstrated^41^ to parallel clinical studies showing mPFC regulation of anxiety responses to an aversive stimulus^13,14^. Increases in PL spine density produced by local α4 knockdown (AAV-Cre) were associated with a 62% decrease in open arm time on the EPM when tested on PND 56 (P = 0.0031, Fig. 6c), and a ~ 40% decrease in open arm time when tested at PND 90 (P = 0.0366, Fig. 6c) in a separate group of animals, compared to AAV-GFP injected control mice. These results suggest that increased spine density in L5 of the PL cortex contributes to increased avoidance behavior, a measure of increased anxiety. However, the number of total entries, a measure of locomotor activity, was not altered by AAV-Cre infusion at either testing age (Fig. 6d).

## Discussion

This study demonstrates that dendritic spine density of L5 PL decreases by half in both female and male adolescent mice due to the emergence of an extrasynaptic GABAR, α4βδ at puberty. Local α4βδ knockdown in the female PL prevented this pubertal pruning and increased anxiety-like behavior in response to an aversive stimulus in late adolescence and adulthood.

Anxiety in the human is associated with excessive avoidance, which maintains the maladaptive fear response^30^. We used the elevated plus-maze to assess the avoidance behavior of mice, which has been verified in humans to reflect anxiety level^30^. This protocol was paired with a mild shock to increase the aversive context to better approximate clinical studies using aversive stimuli to generate mPFC activity in subjects with anxiety^13,14^. An abnormal anxiety response to unpredictable aversive stimuli is a feature of anxiety disorders^8^ which has been studied extensively and is a more revealing outcome than baseline anxiety levels^42^. The post-pubertal anxiety observed after local knockdown of α4βδ in PL at puberty was most likely due to the increase in PL spine density, which is a long-lasting outcome of pubertal α4 knockdown, rather than a result of the decrease in inhibition at puberty because α4βδ expression is low at PND 56 and in adults under control conditions when the behavior is tested. However, the resultant increase in neuronal excitability produced by pubertal α4 knockdown could also increase activation of target sites and potentially alter intracellular messengers in addition to increasing L5 PL spine density.

Anxiety is the most common mental disorder^1^, yet the etiology is not well understood at the circuit level, nor are the potential treatments^10^. This disorder is twice as likely to afflict females, with onset most likely to occur during adolescence^2^ with subtypes ranging from generalized anxiety disorder, agoraphobia, panic disorder, and obsessive–compulsive disorder^43^. These disorders have a high probability of continuing into adulthood^6^ when there is an increased risk of suicide^44^. This study suggests that one contributing factor for anxiety behavior generated post-pubertally is an increase in excitatory synapses in L5 PL via dysregulation of pruning, increasing the input to activate this region.

Excitatory input to L5 PL pyramidal cells comes from the ventral hippocampus, amygdala, and multiple sensory sites^45^. L5 pyramidal cells provide the output of the PL to the basolateral amygdala^46^ to regulate fear and anxiety^12^. Increasing local glutamate concentrations with veratrine in the PL of rodents increases anxiety using the open field test^16^. Blocking NMDARs^47^ in the PL prevents this effect suggesting that anxiety is triggered by NMDAR-mediated transmission. Conversely, numerous studies show that inactivating the PL using either pharmacological or electrolytic techniques reduces anxiety^15,48^. Thus, the present findings correlating L5 PL spine density with avoidance behavior provide a mechanistic link of the PL with increased anxiety. In contrast, the IL is associated with reduced fear/anxiety and fear extinction^12^, due to output to GABAergic neurons via the uncincate fasciculus, which reduces activity in the basolateral amygdala^17^.

Human studies also support a dual role for the PL and IL sub-regions of the mPFC. Dorsal regions of the mPFC, including the anterior cingulate, which corresponds to the rodent PL cortex, are activated by fear^49^. Increased gamma power EEG changes or blood flow accompanies increased fear or anxiety due to fear conditioning or in individuals with generalized anxiety disorder^50–52^. These correlations of enhanced learned fear expression and persistent PL activation are greater in females^49^. In contrast, the human ventromedial PFC (vmPFC), corresponding to the rodent IL, exhibits decreased activity in anxiety^53^. vmPFC lesions increase the amygdala response to aversive stimuli^13^, further confirming the role of the IL/vmPFC in fear reduction.

In the present study, mushroom spines showed the greatest reduction in spine density (74%) in the female L5 PL. The larger head of these spines have a higher density of AMPA receptors^54^ and thus would be expected to have a greater synaptic impact on PL activation. Local α4 knockdown in the PL prevented spine pruning at PND 56, resulting in increased mushroom spine density with levels similar to pubertal wild-type values. Enhanced excitatory transmission to PL would activate output to the amygdala and is a likely mechanism underlying the increased anxiety following local knockdown of α4 expression.

α4 knockdown reversed the 45% decrease in density of the motile spines (thin spines, long thin spines, and filopodia) in adolescence. Motile spines are thought to represent learning spines^55^, which may function in learned fear, such as conditioned cue-related and contextual fear for which the PL plays a role^56^.

α4βδ GABAR expression is altered in the human frontal cortex in some types of mental disorders, especially those that emerge in childhood or adolescence^57^, with decreased expression in brains of non-depressed suicide victims^32,58^. Non-depressed suicide is usually characterized by anxiety^44^. Thus, genetic factors producing dysregulated α4βδ GABAR expression may reduce synaptic pruning during adolescence to increase anxiety.

In cases where there are persistent alterations in expression of α4βδ GABARs, as seen in depression and anxiety^32^, the ultimate effect would depend on the area of expression. Decreased expression of these receptors in the adult prelimbic area would increase anxiety, as suggested by research studies^16^. Increases in α4βδ GABARs are reported in orbitofrontal cortex of suicide victims^31^, which is analogous to the rodent infralimbic. Increased inhibition of this area, outside of the adolescent pruning period, would be expected to increase anxiety, as suggested by clinical imaging studies^13^, and also increase depression, as suggested by studies showing that stimulation of this area is anti-depressant^59–61^.

**Figure 7:**
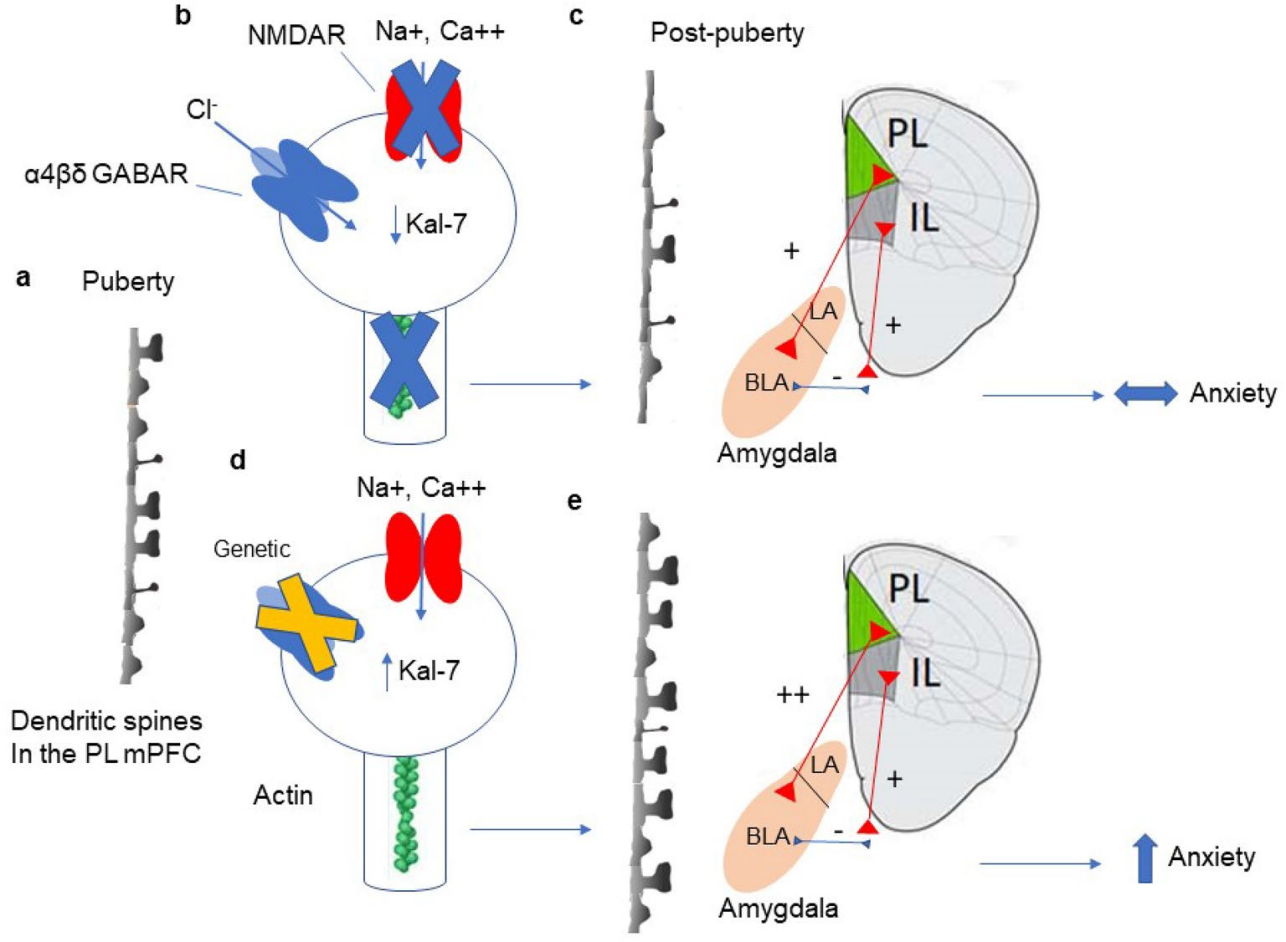
Summary figure. (**a**) Schematic diagram showing representative dendritic spines in L5 PL at puberty. (**b**) Normally, expression of α4βδ GABARs increases on the spine at puberty where they gate a Cl^−^ current that is inhibitory and impairs activation of NMDARs, which gate Na^+^ and Ca^++^ conductances. This decreases Kal-7 expression, resulting in actin de-stabilization, which causes synaptic pruning of L5 PL pyramidal cells. (**c**) The decrease in spine density represents a loss of synaptic input to L5 PL post-pubertally, which reduces excitatory output to the basolateral amygdala. Because this is offset by inhibitory input from the IL, anxiety behavior is unchanged in late adolescence. (**d**) When α4βδ GABAR expression is reduced at puberty (genetically or pharmacologically), NMDARs can generate Kal-7 expression, which stabilizes the actin cytoskeleton, preventing pruning. (**e**) This increased spine density of L5 PL would increase activation of the basolateral amygdala, thereby increasing anxiety in late adolescence. *PL* prelimbic mPFC, *IL* infralimbic mPFC, *LA* lateral amygdala, *BLA* basolateral amygdala.

Increased expression of α4βδ GABARs at puberty was shown both by increases in α4 immunostaining as well as by increased responses of L5 pyramidal cells to the GABA agonist GBX, at a concentration selective for α4βδ GABARs^26^. α4βδ GABAR expression was reduced to near pre-pubertal levels by PND 56, however, suggesting a transient increase in pubertal expression of these receptors. Furthermore, α4 immunostaining was localized to the cell body, dendrites, and the spines at puberty, where these receptors would be expected to impair NMDAR activation, as previously shown^24^ in other CNS areas. The inhibition generated by these receptors along the dendritic shaft as well as on the soma would also impair NMDAR activation by decreasing action potential back-propagation, which is generated in the axon hillock within the soma, travels up the dendrite, and would normally facilitate Mg++ unblock of the NMDAR channel^62–65^. In the present study, increased NMDAR expression generated by administration of low doses of MK-801^38^ during puberty prevented pruning in wild-type mice. In contrast, blocking NMDARs in α4^−/−^ mice using memantine, a treatment which does not increase NMDAR expression^39^, most likely due to its higher affinity for the receptor^40^, restored pruning in the absence of α4βδ-mediated inhibition. These data suggest that α4βδ impairment of NMDARs underlies adolescent pruning of L5 PL. This outcome was mediated by the Rho-guanine nucleotide exchange factor Kal-7, a spine protein necessary for spine maintenance^28^. Kal-7 activates the small GTPase Rac1, which stabilizes the actin cytoskeleton via P21-activated kinases within the spine^66^, and the expression of Kal-7 is increased by NMDAR activation^29^. Thus, decreased Kal-7 expression at puberty would destabilize the spine to enable spine removal. However, Kal-7 expression was increased in L5 PL of pubertal α4^−/−^ mice, suggesting that the increase in α4βδ GABARs in wild-type mice is the initial trigger for the decrease in Kal-7 expression, which leads to pruning, as shown in other CNS sites^29^ (See schematic diagram, Fig. 7). However, we cannot rule out other spine proteins which may play a role in spine stability and pruning^67–69^. In addition, the microglia^70^ and autophagy^71^ have been shown to play a role in pruning but are likely the final steps in this process.

The present findings also show that systemic pubertal administration of the drugs picrotoxin and GBX, which block all GABAR subtypes and potentiate α4βδ GABARs, respectively, was successful in altering PL spine density in the predicted direction at the circuit level. That is, picrotoxin increased spine density, and GBX decreased spine density post-pubertally. This is an interesting finding because the drugs would impact all brain areas, including those with inhibitory inputs to the PL. These findings suggest that pubertal systemic administration of these GABAergic drugs can be used to manipulate spine density in the L5 PL.

In the frontal cortex, synaptic GABAergic afferents target αxβxγ2 GABARs on the dendritic spine^36^. Pubertal administration of the positive GABAR modulator LZM, a benzodiazepine that enhances synaptic inhibition of the dendritic spines at αxβxγ2 GABARs lacking α4^37^, had no significant effect on the overall post-pubertal spine density of the basilar dendrites. This suggests that extrasynaptic α4βδ GABARs, rather than synaptic GABARs, are selectively responsible for synaptic pruning of L5 PL pyramidal cells during adolescence.

Decreases in L5 PL total spine density were > 50% for females across a timespan which reflected puberty onset (~ PND 35) and continued until late adolescence (PND 56). Similar findings were noted for males, which were also due to α4βδ GABARs, as evidenced by the lack of pruning in knock-outs that lacked these receptors' pubertal expression. Synaptic pruning has been demonstrated previously in L5 mPFC, with decreases ranging from < 10% in the rat to 30–40%^21^ in humans for combined IL and PL. A 30% decrease in spine density was reported for combined L3 and L5 PL in male transgenic mice^23^, assessed in early adolescence (PND 31–45), where pubertal timing was not noted. Puberty onset is the time when α4βδ-mediated inhibition increases and triggers pruning; thus, assessments following onset would reflect the greatest change in spine density.

Spine density of L5 PL pyramidal cells ultimately impacts neural networks that generate oscillations with frequencies in the gamma, theta, and delta range^72^. These oscillations represent the emergent properties of recurrent local networks and depend upon the excitatory and inhibitory synaptic input to the dendritic spines of L5 pyramidal neurons. The impedance mismatch between the spine and adjacent dendrite enables the spines to act as coincidence detectors, responding to spatially distributed signals within a limited time window^73^. Thus, spine density determines the sensitivity and reliability of the network to afferent input. In the PL, increased spine density likely results in increased neural activity, which activates downstream targets such as the amygdala and results in increased anxiety. This finding is supported by the present study as well as by clinical imaging studies^74,75^.

In conclusion, α4βδ GABARs were shown to trigger synaptic pruning in L5 PL as an essential process in limiting anxiety responses in late adolescence and adulthood. Dysregulation of pruning increased anxiety responses. These results suggest that deficiencies in the pruning of PL at puberty may be a key physiological mechanism for mental disorders. Given the recent reports showing abnormal gene signals for α4 and δ in some mental disorders^31,32,57,58^, the present findings may suggest therapeutic strategies for anxiety disorders that emerge at puberty.

## Methods

### Animals

For most studies, C57BL/6 wild-type (WT, Jackson Labs) or GABAR α4^−/−^ female and male mice were housed under a reverse light: dark cycle (12:12) and tested in the light phase. α4^−/−^ mice were bred on site from α4 ± mice (supplied by G. Homanics, U. Pitt.). (WT and α4^+/+^ display similar spine densities.) For the Golgi studies, animals were euthanized at the onset of puberty (females, ~ PND35, assessed by vaginal opening; males, ~ PND 37^76^) or PND 56 for spine density analysis. Animals were tested for α4 immunoreactivity and electrophysiological responses pre-pubertally (~ PND 28–32), 1–2 days after the onset of puberty, and post-pubertally (PND 56). The estrous cycle is not a factor during the pubertal period (PND 35–44)^77,78^. However, the estrous stage was determined for animals euthanized on PND 56 using vaginal smears in order to avoid the stage of proestrus when GABAR expression and dendritic spine counts can be increased^79,80^. For drug administration studies, all animals were injected once daily (intraperitoneally) with the following drugs from PND 35 (onset of puberty) to PND 49, the period of high α4 expression: gaboxadol (GBX, THIP, 4,5,6,7-tetrahydroisoxazolopyridin-3-ol), 0.1 mg/kg, a dose which has no effect in α4^−/−^ mice^35^; picrotoxin, 3 mg/kg; lorazepam (LZM), 0.25 mg/kg; MK-801 ([5R,10S]-[+]-5-methyl-10,11-dihydro-5H-dibenzo[a,d]cyclohepten-5,10-imine), 0.1 mg/kg, a dose which increases NMDAR expression in mPFC^38^; memantine (1-Amino-3,5-dimethyladamantane), 10 mg/kg, an NMDAR blocker which does not increase NMDAR expression^39^. In all experimental procedures, mice were randomly assigned to experimental groups, and the investigator was blinded to the condition of the mice. All procedures were approved by the SUNY Downstate Medical Center institutional animal care and use committee and carried out in accordance with their guidelines and regulations. In addition, the authors complied with the ARRIVE guidelines.

### Local knockdown of the GABAR α4 subunit

Transgenic female mice with loxP (locus of X-over P1) sites flanking the α4 gene (B6.129-GABRA4^tm^^1^^.2Geh^/J) were purchased from Jackson Labs (Bar Harbor, ME) and bred on site to yield homozygous offspring (genotyping by Transnetyx, Cordova, TN). Local α4 knockdown procedures were undertaken on the PND 21 female mice. Following induction of anesthesia using a cocktail, injected intraperitoneally, of ketamine (75 mg/kg) with dexmedetomidine (0.5 mg/kg), the mice were placed in a stereotaxic apparatus. Mice were locally infused with 0.25 μl of either adeno-associated virus-Cre recombinase with green fluorescent protein (AAV-Cre/GFP, pAAV.CMV.HI.eGFP-Cre.wPRE.SV40, ≥ 8 × 10^12^ vg/μl, cat# 105545-AAV1) or AAV-GFP (pAAV-CAG-GFP, cat# 37825-AAV5) into the prelimbic region of the mPFC (coordinates: AP 1.9, ML ± 0.3, DV − 1.45), bilaterally, using an infusion pump and a Hamilton syringe (flow rate: 0.12 μl/min). Both viral constructs were from Addgene (Watertown, MA). The surgical site was sutured, and animals were allowed to recover for 2 weeks but returned to group housing after 48 h. In some cases, viral entry and selective PL targeting were verified using Cre and/or GFP immunohistochemistry, respectively, at PND 35–37. Successful α4 knockdown was determined using α4 immunohistochemistry in the Cre-injected mice at PND 35–37, compared to GFP-injected controls, when puberty onset was also determined. In other cases, mice were either euthanized to assess spine density using Golgi procedures (PND 56) or tested for anxiety using the elevated plus-maze (EPM, PND 56–68, 90–111) followed by confirmation of α4 knock-down.

### Immunohistochemistry

Following anesthesia with urethane (0.1 ml 40%), mice were perfused with saline (12–15 ml/min) and then with 4% paraformaldehyde (PFA) followed by post-fixation of the brain in 4% PFA (48 h, 4 °C).

#### Paraffin-embedded sections

PFA preserved brains were embedded in paraffin blocks following tissue dehydration using increasing ethanol concentrations. Coronal sections of the mPFC were cut on a microtome at a thickness of 10 μm and mounted on super-frost slides. Tissue was de-paraffinized in decreasing concentrations of ethanol and processed using antigen retrieval: Slides were incubated in warm (95–100 °C) sodium citrate buffer (10 mM sodium citrate, 0.05% Tween 20, pH 6.0) for 30 min, allowed to cool, and rinsed (2×) with 0.01 M phosphate-buffered saline (PBS), 0.05% Tween 20 (PBS-Tween) for 2 min.

#### Free-floating sections

Coronal sections of the mPFC were cut on a vibratome (Leica VT 100 M) at a thickness of 30–40 μm. Free-floating sections were washed (3x) in PBS-Tween with 1% bovine serum albumin (BSA) for 10 min.

#### Immunohistochemistry protocol

Sections were blocked in PBS supplemented with 1.5% donkey serum (kalirin) or 1.5% goat serum (α4, Cre) in PBS-Tween 2 h at room temperature. α4: Sections were incubated in blocking buffer containing 2% goat anti-mouse Fab fragments (Jackson Immunolabs, Bar Harbor, ME) for 2 h at room temperature. Then, sections were incubated with anti-α4 (mouse monoclonal, Antibodies, Inc., Davis, CA, 1:100). In some cases, anti-α4 (goat polyclonal, sc7355, Santa Cruz, 1:20) with anti-MAP2 (microtubule-associated protein-2, ab5392, Abcam, Cambridge, MA, 1:1000) were used without pre-incubation with the anti-mouse Fab fragments to verify α4 localization on dendritic spines. Both antibodies show selectivity for α4 as evidenced by their lack of staining in the hippocampus of α4 knock-out mice shown here (Supp. Figure 7) and in a previous publication^81^. Although MAP2 is localized to the soma and dendrites, it can also be localized to spines and has been used as a spine marker^82–84^. MAP2 is primarily localized to mushroom spines^33^ which are one of the predominant spine types at puberty. Therefore, we used MAP2 to visualize dendrites and spines at puberty.

#### Kalirin, Cre, NMDAR1

Anti-kalirin-7 (Kal-7, rabbit polyclonal, a generous gift from R Mains, UConn Health, JH2958^85^, 1:200), anti-Cre (rabbit polyclonal, Novus Biologicals, Centennial, CO, 1:1000) or anti-NMDAR1 (rabbit monoclonal, ab274377, Abcam, Cambridge, MA, 1:100) were used. General: All antibodies were diluted in the blocking solution and incubated with tissue sections overnight at 4 °C. After washing, sections were incubated with the appropriate fluorescent secondary antibody (Alexa fluor 488 and 594, 1:1000) for 2 h, washed in PBS 3× for 10 min, after which they were mounted on slides with ProLong Glass antifade reagent in some cases with 5% nuclear blue. Images were taken with an Olympus FluoView TM FV1000 confocal inverted microscope with objective UPLSAPO 40× or 100× NA:1:30 (Olympus, Tokyo, Japan). For the immunohistochemical analysis, the merged z-stack image (2 μm steps) was used. Image segmentation was first performed using a thresholding sub-routine in ImageJ so that the original color image was converted to a binary image. This allowed for visualization of the regions of interest (ROI) in cases where the background intensity was non-homogeneous. ROIs were then analyzed for image luminosity in the original image using Adobe Photoshop after subtracting the adjacent background levels, and the results were verified by ImageJ. 3 ROIs were analyzed per mouse.

### Golgi procedure

Before euthanization, mice were anesthetized with urethane (1–2 g/kg, i.p.)^24^, and whole brains were extracted and processed for Golgi impregnation with the FD Neurotechnologies Rapid Golgi Stain kit. Coronal sections were prepared using a vibratome (Leica VT1200s) set to a thickness of 250 μm.

#### Analysis

Pyramidal cells from L5 PL were identified using The Mouse Brain in Stereotaxic Coordinates (4th Edition, Paxinos, and Franklin, 2012) and the Allen Brain Institute’s Mouse Brain Atlas (http://mouse.brain-map.org). The L5 PL neurons were approximately 1.7 mm ventral from the dorsal surface and the cell bodies were 500–700 μm from the medial surface. Individual neurons in these regions were viewed using a 100× oil objective on a Nikon Eclipse Ci-L microscope. Images of the basilar dendrites were acquired using Z-stack projection photomicrographs (0.1–0.9 μm steps) taken using a Nikon DS-U3 camera mounted on the microscope and were analyzed using NIS-Elements D 4.40.00 software. Three to four neurons (middle 80%) were sampled per mouse, and six segments in the same field of view were assessed per neuron (20–50 μm). Each dendrite segment was ~ 1 μm thick and was taken from a 2° or 3° order dendrite. Spine density was expressed as the number of spines/10 μm. To determine the type of dendritic spine, we used parameters described by Risher et al.^86^: filopodia, length > 2 µM; long thin, length < 2 µM; thin, length < 1 µM, stubby, width ratio < 1 µM, mushroom, width > 0.06 µM; bifurcated, two or more heads.

### Electrophysiology

#### Cortical slice preparation

Brains from euthanized mice were removed and cooled using an ice-cold solution of artificial cerebrospinal fluid (aCSF) containing (in mM): NaCl 124, KCl 2.5, CaCl_2_ 2, NaH2PO_4_ 1.25, MgSO_4_ 2, NaHCO_3_ 26, and glucose 10, saturated with 95% O_2_, 5% CO_2_ and buffered to a pH of 7.4. Following sectioning at 400 μm on a Leica VT1000S vibratome, slices were incubated for 1 h in oxygenated aCSF.

#### Cortical slice voltage-clamp electrophysiology

Pyramidal cells in L5 PL were visualized using a differential interference contrast (DIC)-infrared upright Leica microscope and recorded using whole-cell patch clamp procedures in voltage clamp mode at 26–30 °C, as described^77^. Patch pipets were fabricated from borosilicate glass using a Flaming-Brown puller to yield open tip resistances of 2–4 MΩ. For recordings of the pharmacologically isolated tonic inhibitory current, the pipet solution contained in mM: CsCl 140, HEPES 5, EGTA 5, CaCl2-H2O 0.5, QX-314 5, Mg-ATP 2, Li-GTP 0.5, pH 7.2, 290 mOsm. 5 mM QX-314 was added to block voltage-gated Na+ channels and GABA_B_ receptor-activated K+ channels. The aCSF contained 50 μM kynurenic acid to block excitatory current, as well as 0.5 μM TTX to isolate the post-synaptic component. Recordings were carried out at a − 60 mV holding potential, and the tonic current was assessed by the change in holding current in response to 100 nM gaboxadol (GBX), a GABAR agonist which, at this concentration, is selective for δ-containing GABAR^26,34^. The GABAergic nature of the current was verified by block with 100 μM picrotoxin. Drugs were bath applied continuously in sequential order following 5–10 min of baseline recordings without drugs. Recordings were conducted with a 2 kHz 4-pole Bessel filter at a 10 kHz sampling frequency using an Axopatch 200B amplifier and pClamp 9.2 software. Electrode capacitance and series resistance were monitored and compensated; access resistance was monitored throughout the experiment, and cells were discarded if the access resistance increased more than 10% during the experiment. In all cases, the data represent one recording/animal.

### Anxiety response to an aversive stimulus assessment using avoidance behavior

Mice were tested for anxiety-like behavior using the shock-paired elevated plus maze (EPM), an established model of anxiety^41,87^, which assesses avoidance behavior, on PND 56 or PND 90 following local α4 knockdown at puberty in response to AAV-Cre infusion on PND 21. Local knock-down was verified with immunohistochemical techniques after the behavioral test. We tested anxiety in response to an aversive stimulus to mimic human studies, which show mPFC regulation of anxiety in response to aversive settings^13,14^. Results were compared with the GFP control (AAV-GFP infusion on PND 21). The plus-maze consists of four 8 × 35 cm arms at 90° angles, elevated 57 cm above the floor (Fig. 7c inset). 33 cm walls enclose two arms, and two arms have no walls (“open arms”). The open arms are also partially bordered by small rails (5 × 15 cm) extending to the proximal half of the arm, and the floor of the maze is marked with grid lines every 25 cm. Each animal was initially acclimated to the room for 30 min–1 h. Then, mice were administered a 400-μA shock for 1 s^17^ immediately before being placed in the maze center when exploratory activity was recorded for 5 min. The time spent in the open and closed arms was tabulated, as were the entries. To be considered an open arm entry, the animal had to cross the open platform's line with all four paws. A decrease in open arm time is considered a measure of increased avoidance behavior, reflecting anxiety^87^, as we have described^41^. The number of total entries is a measure of general activity level.

### Drugs

All drugs except QX-314 were from Sigma Chemical Co (St Louis, MO). QX-314 was from Calbiochem (Billerica, MA).

### Statistics

Statistics were analyzed with Prism-GraphPad (spine densities) or OriginPro (all other data). Data are presented as the mean ± S.E.M., and in some cases, the median, interquartile range, and outliers are indicated. Individual data points are presented when n < 10. Data were shown to have similar variance using the Brown-Forsythe test for equal variance and were verified as reflecting a normal distribution by the Kolmogorov–Smirnov test. The significant differences in spine densities calculated across treatment groups were analyzed with a nested t-test (2 groups) or a nested one-way analysis of variance (ANOVA, > 2 groups) with a post-hoc Tukey test (male data) or Dunnett’s test (pharmacology study). Averaged values calculated across treatment groups for immunohistochemistry, electrophysiology and behavior were analyzed with the Student’s t-test (2 groups) or one-way analysis of variance (ANOVA, > 2 groups) with a post-hoc Tukey test for unequal replications. All tests were two-tailed. A *P* value < 0.05 was used as an indication of statistical significance. A power analysis was conducted to determine adequate sample size for all studies, which achieved a power > 0.85. Reproducibility was determined by comparing the statistical significance of results from experiments performed 3–5 times to achieve the final n’s.

## Supplementary Information

Supplementary Figures.

## Data Availability

Source data files for all experiments are available upon request.
