# Supplementary material for: Preventing adolescent synaptic pruning in mouse prelimbic cortex via local knockdown of α4βδ GABAA receptors increases anxiety response in adulthood
Source: Sci Rep. 2021 Oct 26;11:21059. doi: 10.1038/s41598-021-99965-8 (PMC8548505; doi:10.1038/s41598-021-99965-8)
Supplement: Supplementary file 1 — Supplementary Figures. [file 41598_2021_99965_MOESM1_ESM.pdf]

# Preventing adolescent synaptic pruning in mouse prelimbic cortex via local knockdown of α4βδ $GABA_A$ receptors increases anxiety response in adulthood

*Matthew R. Evrard, Michael Li, Hui Shen and Sheryl S. Smith*

## Supplementary material

### Supplementary figure 1.

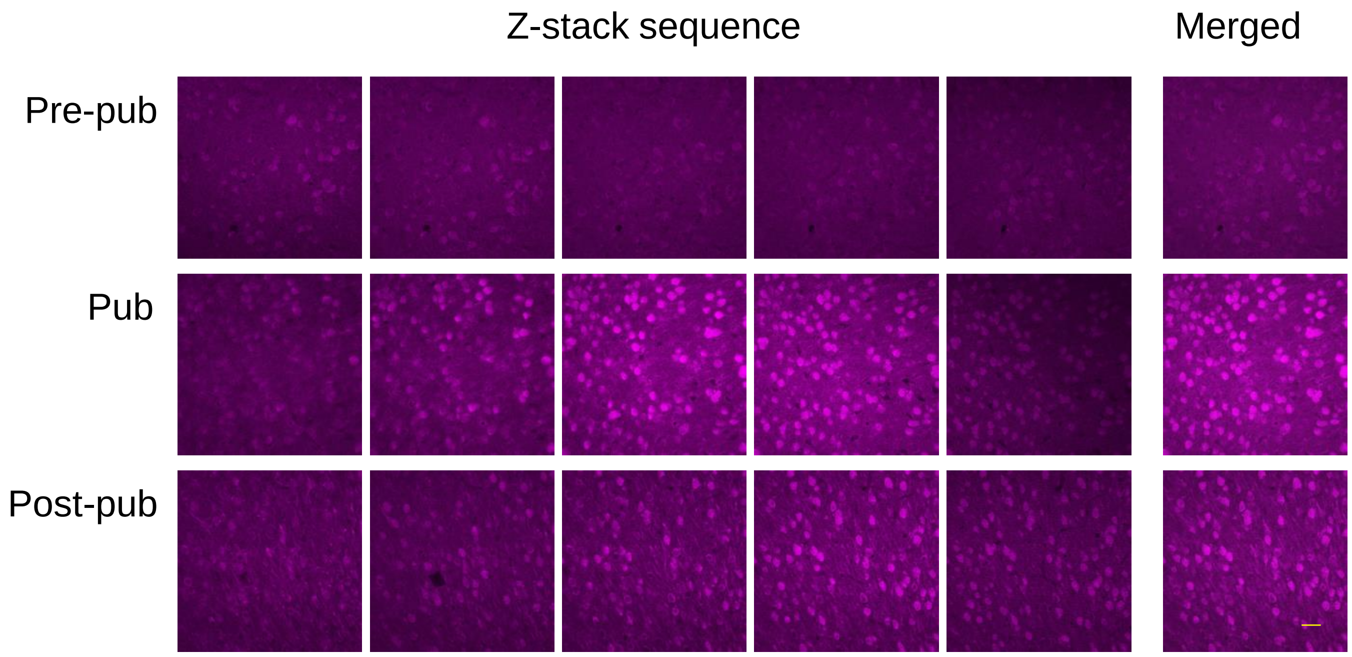


**Z-stack sequences of α4 immunostaining in prelimbic cortex of female mice across adolescence**

Left, Representative images taken at 2 μm z-steps of α4 immunostaining in prelimbic cortex from pre-pubertal (Pre-pub, top), pubertal (Pub, after vaginal opening, ~PND 35, middle) or post-pubertal (Post-pub, bottom) female mice. Right, Merged z-stack images. Statistics are presented in Fig. 2. Scale 100 μm.

Supplementary figure 2.

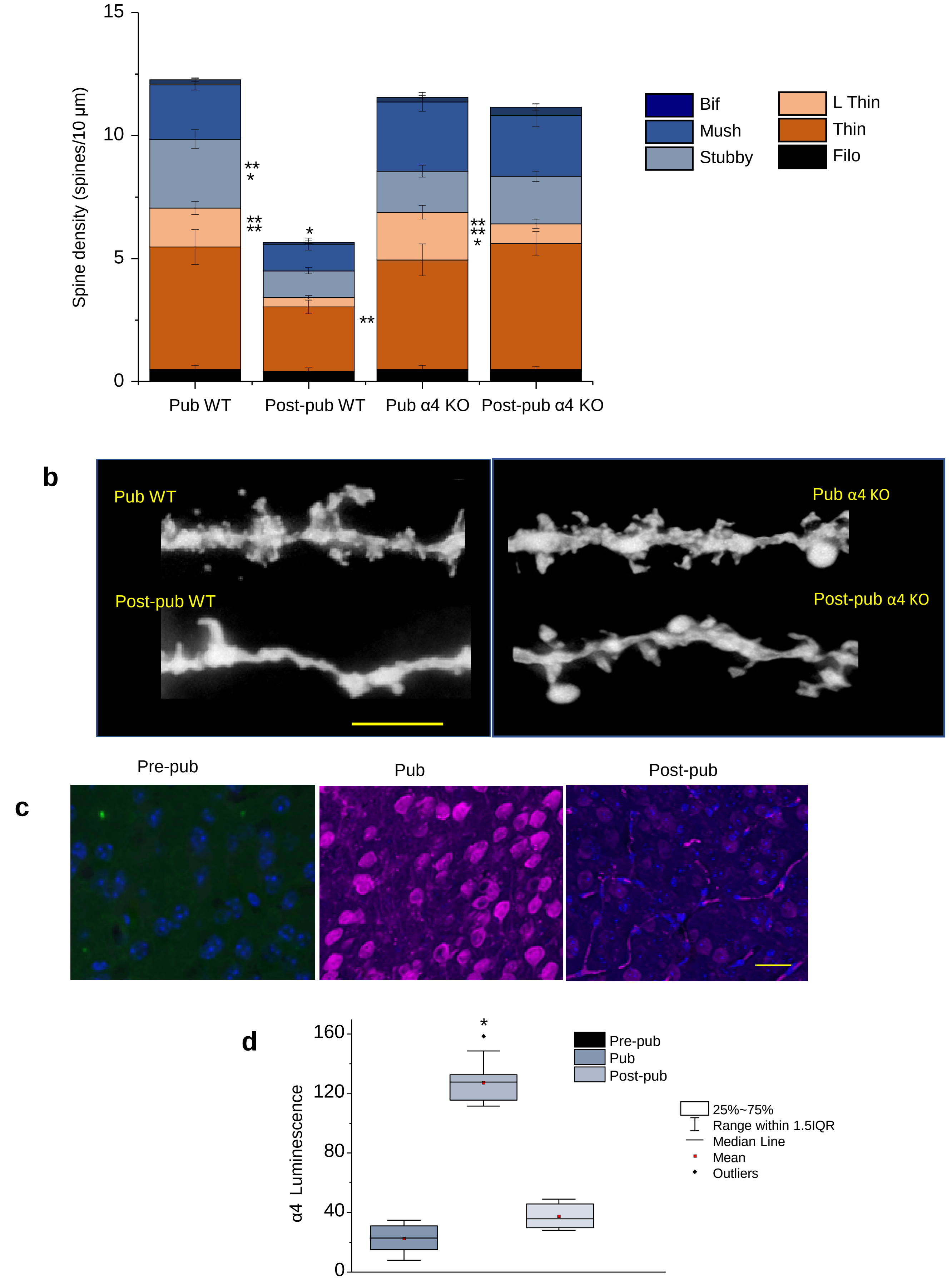

**Supplementary figure 2. Synaptic pruning in male L5 PL is dependent upon pubertal increases in α4βδ GABAR expression.**

a, Averaged data, spine density (#spines/10 μm) of pubertal (Pub) and post-pubertal (Post-pub) wild-type (WT) and α4 -/- male mice. Total spines, $F(3,32)=13.76$, *P<0.0001[Pub WT vs. Post-pub WT, P<0.0001; Post-pub WT vs. Pub α4-/-, *P<0.0001; Post-pub WT vs. Post-pub α4-/-, *P=0.0007]; mushroom, $F(3,32)=5.28$, *P=0.0045 [Pub WT vs. Post-pub WT, P=0.0842; Post-pub WT vs. Pub α4-/-, *P=0.0039; Post-pub WT vs. Post-pub α4-/-, *P=0.0282]; stubby, $F(3,32)=8.05$, *P=0.0004 [Post-pub WT vs. Pub WT, *P=0.0002; Pub WT vs. Pub α4-/-, *P=0.0159]; long thin $F(3,32)=10.41$, *P<0.0001 [Post-pub WT vs. Pub WT, *P=0.0027; Post-pub WT vs. Pub α4-/-, *P<0.0001; Pub α4-/- vs. Post-pub α4-/-, *P=0.0076]; thin, $F(3,32)=4.22$, *P=0.0127 [Post-pub WT vs. Pub WT, *P=0.0222; Post-pub WT vs. Post-pub α4-/-, *P=0.0246]. *P<0.05 vs. other groups; **P<0.05 vs. pubertal groups, n=25-36 neurons, 9 mice/group. b, Representative images of basal dendrites from Golgi-stained neurons, from Pub and Post-pub male WT and α4 -/- mice. Scale, 5 μm. c, Representative images, α4 immunostaining (magenta), L5 PL pyramidal cells from Pre-pub (left), Pub (center) and Post-pub (right) male mice (α4, magenta; DAPI, blue). Scale, 25 μm. d, Averaged data, mean, median and interquartile range (IQR). $F(2,42)=468.31$, P<0.00001. *P<0.05 vs. other groups. n=15 neurons, 10 mice/group.

# Supplementary figure 3.

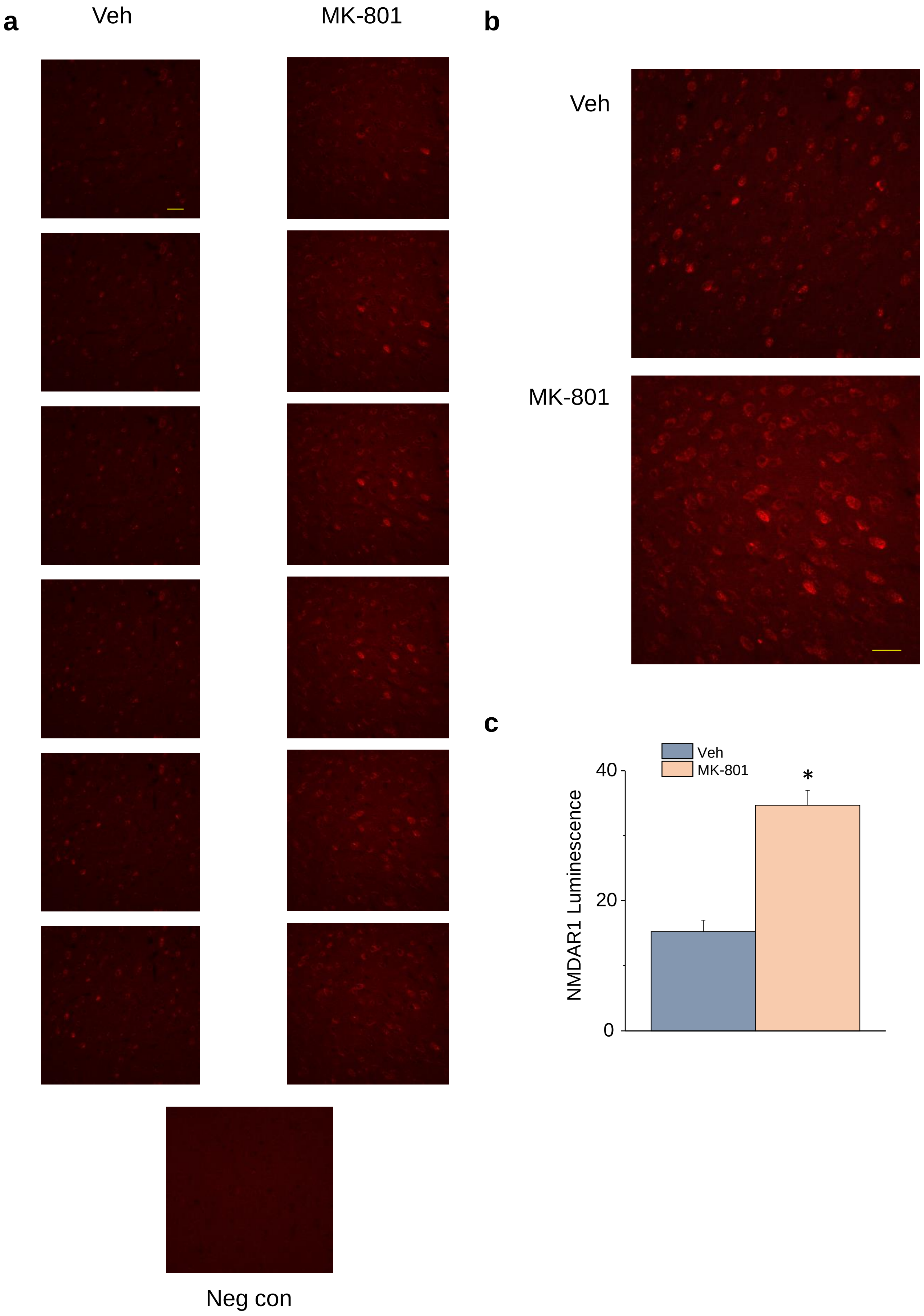


**Pubertal MK-801 administration increases NMDAR1 expression in prelimbic cortex.** a) Representative images taken at 2 μm z-steps of NMDAR1 immunostaining in prelimbic cortex from mice treated with vehicle (Veh, left) or MK-801 (right) beginning at the onset of puberty (vaginal opening, ~PND35) for 5 days. Neg control, immunostaining assessed without the primary antibody. Scale, 100 μm. b) Merged z-stack images. Scale 100 μm. c) Averaged data. MK-801 increased NMDAR1 expression predominantly in the soma and proximal dendrites as reported[1]. *$t(18)=6.8$, $P<0.00001$ versus control. n=10 neurons, 5 mice.


1 Dodt, H. U., Frick, A., Kampe, K. & Zieglgansberger, W. NMDA and AMPA receptors on neocortical neurons are differentially distributed. *Eur J Neurosci* **10**, 3351-3357, doi:10.1046/j.1460-9568.1998.00338.x (1998).

# Supplementary figure 4.

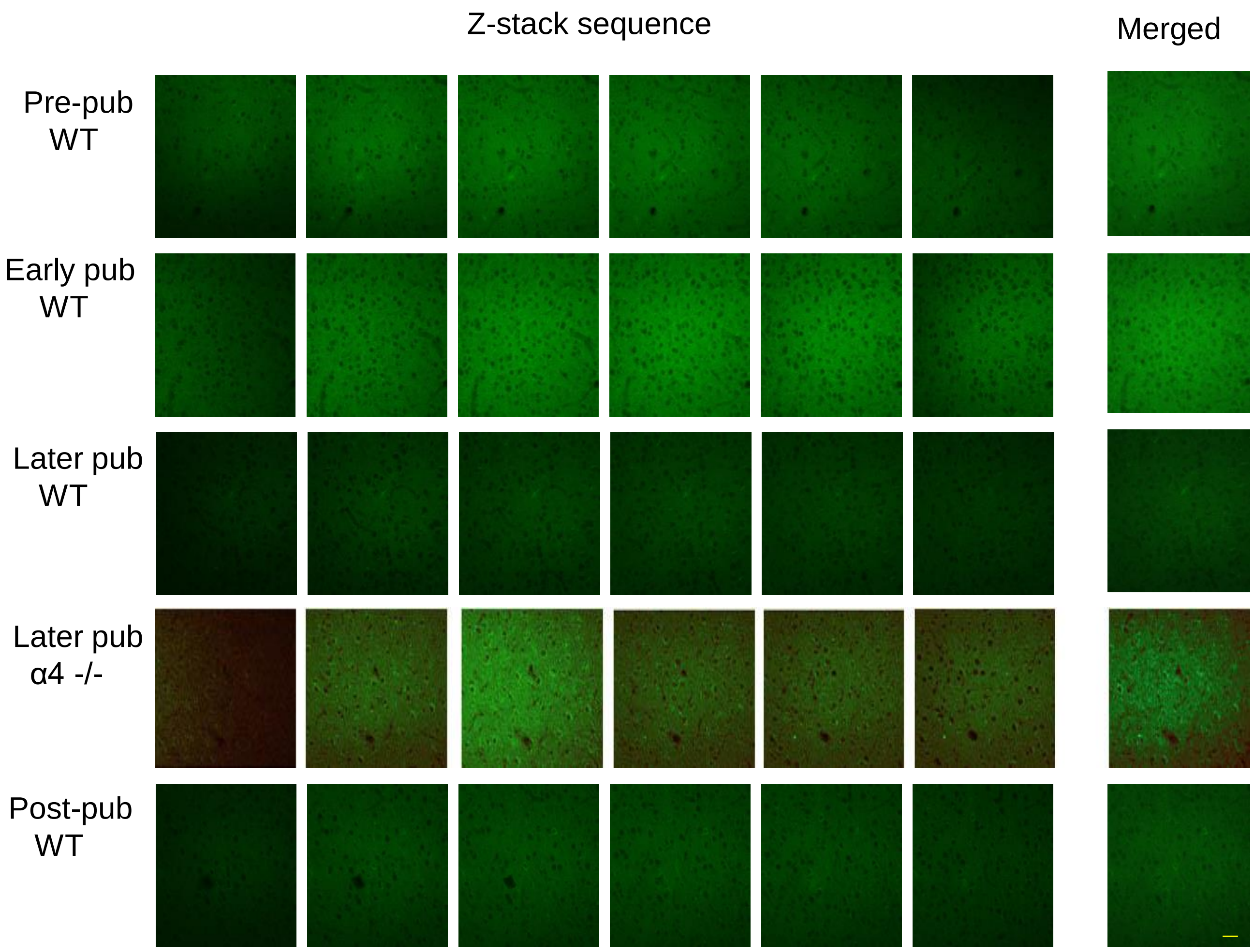


**Z-stack sequences of Kalirin-7 immunostaining in prelimbic cortex of female mice across adolescence**

Left, Representative images taken at 2 μm z-steps of kalirin-7 (Kal-7) immunostaining in prelimbic cortex from pre-pubertal wild-type (Pre-pub WT), early pubertal wild-type (Early pub WT, the day of vaginal opening, ~PND 35), later pubertal wild-type (Later pub WT, after vaginal opening, PND 36-40), later pubertal α4 -/- (Later pub α4 -/-) or post-pubertal wild-type (Post-pub WT, PND 56) female mice. Right, Merged z-stack images. Statistics are presented in Fig. 4. Scale, 100 μm.

Supplementary figure 5.

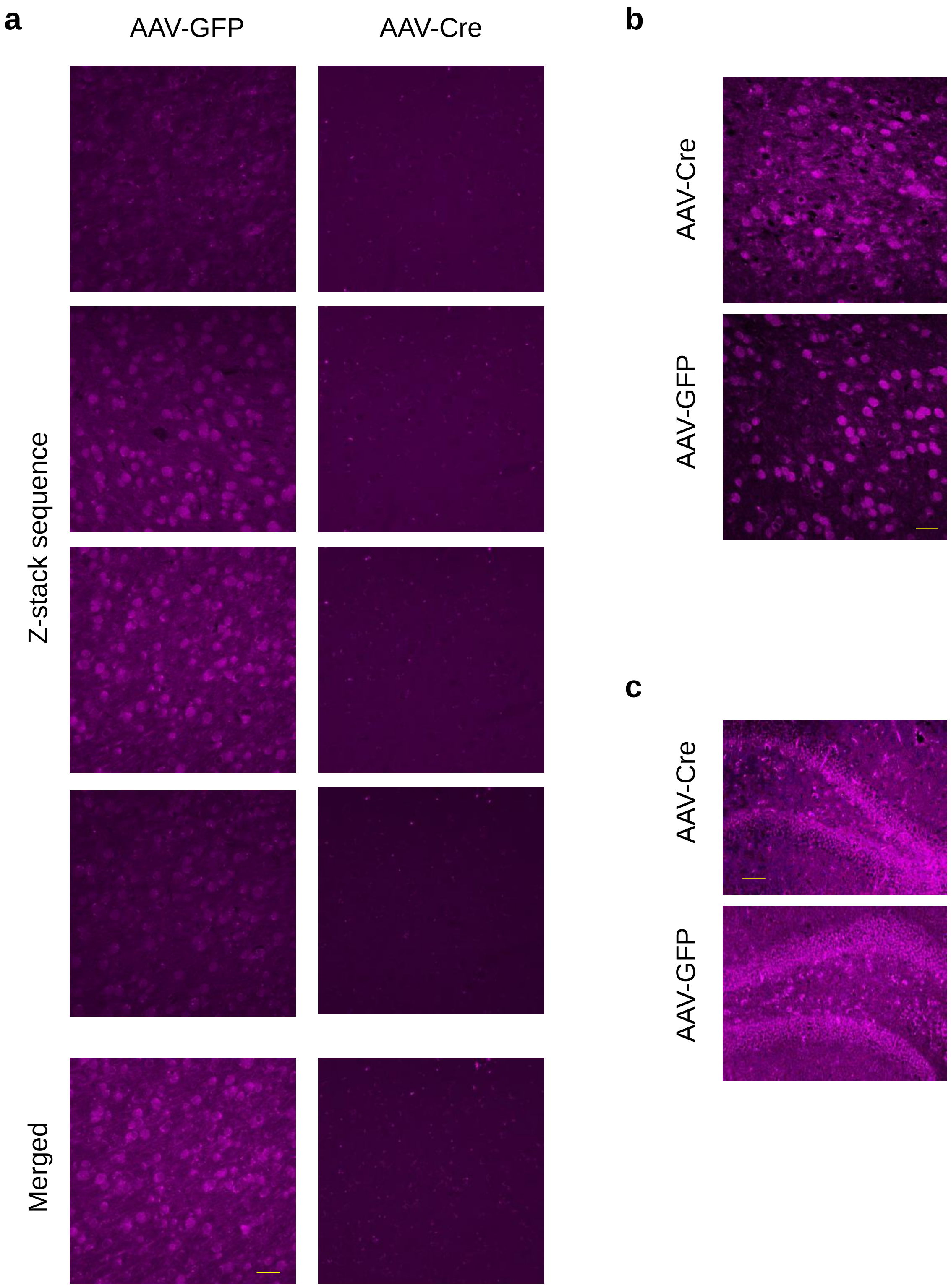


**Localized α4 knock-down in prelimbic cortex after AAV-Cre infusion: Z-stack sequences**

a, Top, Representative images taken at 2 μm z-steps of α4 immunostaining in prelimbic cortex from pubertal mice locally infused with AAV-GFP (left) or AAV-Cre (right) to knock-down α4 on PND 21. Bottom, Merged z-stack images. Statistics are presented in Fig. 5. Scale 100 μm. b, Representative images, α4 immunostaining in infralimbic cortex from mice infused with AAV-Cre (top) or AAV-GFP (bottom). Scale 100 μm. c, Representative images, α4 immunostaining in dentate gyrus from mice infused with AAV-Cre (top) or AAV-GFP (bottom). Scale 200 μm. α4 was selectively knock-down in the prelimbic cortex, but not infralimbic or dentate gyrus.

# Supplementary figure 6.

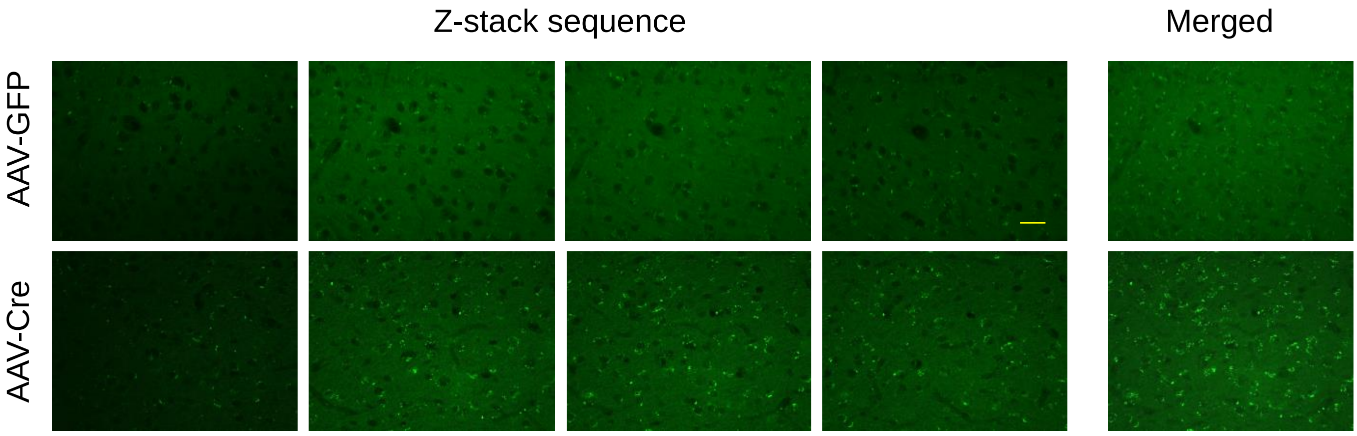


**Z-stack sequences of α4 immunostaining in prelimbic cortex of female mice following local knock-down of α4**

Left, Representative images taken at 3 μm z-steps of kalirin-7 (Kal-7) immunostaining in prelimbic cortex from mice locally infused with AAV-GFP (top) or AAV-Cre (bottom) to knock-down α4 on PND 21. Right, Merged z-stack images. Statistics are presented in Fig. 5. Scale 100 μm.

## Supplementary figure 7.

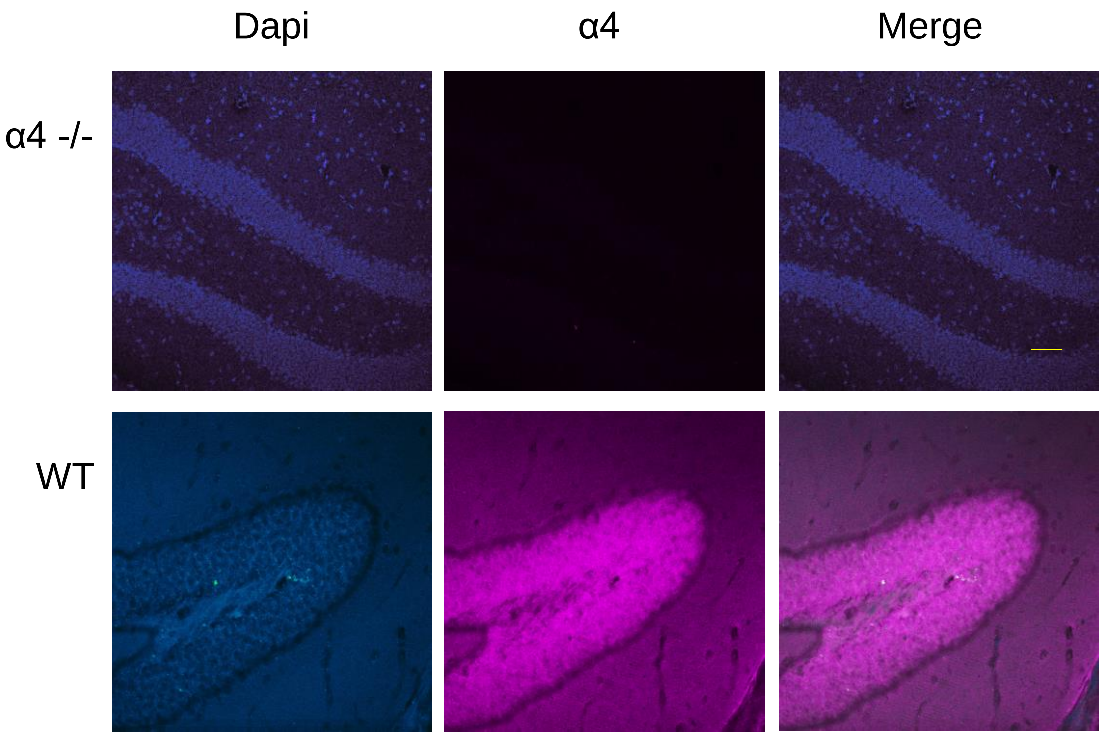


**Specificity of α4 immunostaining.**

α4 staining in dentate gyrus of an α4 -/- (upper panel) and wild-type (WT) mouse (lower panel). Left, Dapi staining; Middle, α4 staining; Right, merged. Staining is only evident in the slice (WT) containing α4-$GABA_A$ receptors, reflecting high antibody specificity. Scale 100 μm.
